# Necrotizing fasciitis in the orbital and submandibular areas of the right face: a rare clinical image

**DOI:** 10.11604/pamj.2024.47.104.42758

**Published:** 2024-03-05

**Authors:** Emmanuel Lalrinchhana, Deeplata Mendhe

**Affiliations:** 1 Department of Community Health Nursing, Srimati Radhikabai Meghe Memorial College Of Nursing, Datta Meghe Institute of Higher Education and Research, Sawangi, Wardha, Maharashtra, India

**Keywords:** Facial necrosis, necrosis, necrotizing fasciitis, flesh eating disease, deformity

## Image in medicine

Necrotizing means the process of dying tissues or deterioration of the tissue. Inflammation of the fascia is referred to as fasciitis. It is a sporadic infection caused by a bacterium that spreads promptly throughout the body, and it could be tragic and terminal. Necrotizing fasciitis is typically caused by group A Streptococcus, according to public health experts (group A strep). A 58-year-old diabetic male patient was brought with a case of uncomfortable swelling on the right submandibular and orbital areas for 3-5 days followed by a portion of the orbital region displayed scabbing of necrosed skin, and the overlying skin was oedematous and reddish. Also, he was reported having excruciating facial pain, mostly in the orbital area after some time, and had to have facial surgery to remove the necrosis-affected parts, which are primarily in the orbital region of the right face, because his condition persisted after the treatment and did not improve for a while. The presence of fascial necrosis during surgery and the analysis of tissue samples revealed group A streptococcal infection, which later validated the diagnosis. For medical intervention, the patient was treated with injection Piperacillin and Tazobactam 4.5 gram I/V Q8H, injection Pantoprazole 40mg I/V x OD, injection Ondansetron 4mg x Q8H and injection tramadol 100mg in 100ml normal saline I/V x BD. Surgical intervention- debridement with complete removal of the necrosed skin. Necrotizing fasciitis is a rare illness but one that has a high risk of significant morbidity, without surgery, mortality is very close to 100%.

**Figure 1:**
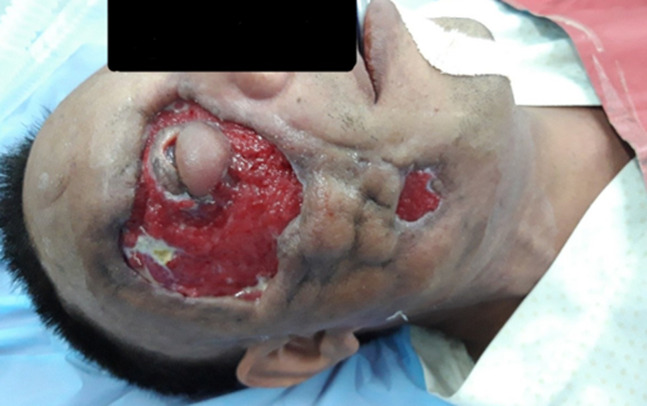
necrotizing fasciitis in the orbital and submandibular areas of the right face

